# Testing Species Delimitations in Four Italian Sympatric Leuciscine Fishes in the Tiber River: A Combined Morphological and Molecular Approach

**DOI:** 10.1371/journal.pone.0060392

**Published:** 2013-04-02

**Authors:** Lorenzo Tancioni, Tommaso Russo, Stefano Cataudella, Valentina Milana, Anne Kathrin Hett, Elisa Corsi, Anna Rita Rossi

**Affiliations:** 1 Experimental Ecology and Aquaculture Laboratory, Biology Department, “Tor Vergata” University of Rome, Rome, Italy; 2 Biology and Biotechnology Department, “La Sapienza” University of Rome, Rome, Italy; University of Lausanne, Switzerland

## Abstract

Leuciscine fishes represent an important component of freshwater ichthyofauna endemic to northern Mediterranean areas. This lineage shows high intra-specific morphological variability and exhibits high levels of hybridization, two characteristics that contribute to systematic uncertainties, misclassification of taxa and, potentially, the mismanagement of biodiversity. This study focused on brook chub, *Squalius lucumonis*, an endemic taxon of Central Italy. The taxonomic status of this species has long been questioned, and a hybrid origin from sympatric leusciscines (*S. squalus* x *Rutilus rubilio*, or *S. squalus* x *Telestes muticellus*) has been hypothesised. A phenotypic (evaluating shape and meristic counts) and genetic (using mitochondrial and nuclear markers) investigation of these four taxa was conducted to test species delimitation in sympatric areas and to evaluate the taxonomic status of *S. lucumonis*. One hundred and forty-five individuals of all four taxa were collected within streams of the lowest portion of the Tiber River basin and analysed; this region encompasses a large portion of the *S. lucumonis* distribution. The different morphological and genetic approaches were individually examined, compared, and then combined in a quantitative model to both investigate the limits of each approach and to identify cases of misclassification. The results obtained confirm the cladogenetic non-hybrid origin of *S. lucumonis*, highlight the need for immediate conservation actions and emphasise the value of an integrated approach in the study of leuciscines evolution.

## Introduction

Numerous cases of hybridization and introgression events have been found by molecular studies in many organisms. The occurrence of such events makes the biological and other (e.g the phylogenetic) species concepts difficult to be applied univocally [1]. Most of the reported cases of hybridization in animals involve fishes, mainly freshwater species [1]. Among these, cyprinids are known to exhibit high rates of hybridization at both the intra- and intergeneric level. This is especially evident for leuciscine species [2]–[9]; approximately 62 different intra- and intergeneric hybrids were described for this group in the wild [10]. Leuciscine fishes were traditionally classified as a subfamily (Leuciscinae) within the Cyprinidae family [11], [12]. However, based on molecular data, Chen & Mayden [13] proposed that these fishes, along with nine other monophyletic groups of cyprinid, be classified each as a family within the superfamily Cyprinoidea. Thus, according to these authors, Leuciscinae should be considered as Leuciscidae.

In Europe, Cyprinoidea are dominant [14] and comprise a large number of endemic species in northern Mediterranean regions [15], including Italy [16]. Many leuciscine fishes are listed in the International Union for Conservation of Nature (IUCN) Red List [17], represent many of the threatened freshwater fishes of Europe [18] and are listed in Annex II of the European Union Habitats Directive 92/43/EEC (http://ec.europa.eu/environment/nature/legislation/habitatsdirective) and in Appendix III of Bern Convention [19], [20]. Moreover, many leuciscine species distributed within the Mediterranean region show high intraspecific morphological variability [21]. The described aspects of Leuciscidae biology make it difficult to resolve the phylogenetic and taxonomic status of several populations and species in the Mediterranean area. Thus efforts should be made to validate the taxonomy of this family and to identify diagnostic characters for species identification.

Over the last decade, multidisciplinary approaches have proven useful in cases of unclear taxonomic status that are very common when species tend to hybridize [22]–[24]. Resolving taxonomic uncertainties is particularly important for the identification of conservation units and relevant management strategies [25]. Combined morphological and genetic analysis have been successfully applied to resolve taxonomy [7], [26]–[29] and to interpret ecomorphology [9] within this family.

In the present study, morphological and molecular data were used mainly to investigate an Italian leuciscine, the brook chub *Squalius lucumonis*. This species is endemic to the Tuscano-Latium district of Central Italy. Its distribution appears to be restricted to the Tyrrhenian drainage basins of Ombrone-Serchio, Arno and Tiber [30]–[32], with approximately 12 main populations estimated [30]. Its first description by Bianco [33] was based exclusively on morphologic and meristic (countable) traits derived from the analysis of a few individuals caught within the Tiber and Ombrone River basins. The taxonomic status of *S. lucumonis* was questioned by Gandolfi et al. [34] and Zerunian [35], who argued that the brook chub might represent a hybrid between *S. squalus* (previously included in the *S. cephalus* taxon) and another sympatric species of Leuciscinae. Based on meristic characters, these authors proposed *S. squalus* x *T. muticellus* (previously included within the *T. souffia* complex) or *S. squalus* x *R. rubilio* as the potential parental species crosses. Manaresi et al. [36] rejected the hypothesis of a hybrid origin based on the absence of heterozygosity at diagnostic allozyme loci and at total protein bands between the presumed parental species. Furthermore, they found alternative alleles at one locus and the presence of two diagnostic bands (65 kDa and 26 kDa) in total protein analysis between *S. lucumonis* and *S. squalus*, and proposed that the two taxa may have diverged recently from a common ancestor.

Finally, morphological brook chub-like individuals were recorded in an area outside the natural range of *S. lucumonis;* genetic analysis identified these individuals as hybrids between *S. squalus* and *R. rubilio* [37]. This paper seeks to evaluate the morphological variation and taxonomic status of *S. lucumonis* in relation to three other sympatric Italian leuciscines: *S. squalus,* also distributed along the Dalmatian coasts, *T. muticellus,* also present along the south French-Italian border and in Switzerland, and the Italian endemic *R. rubilio* [30]. The latter two species are listed as endangered in Annex II of the European Habitat Directive [38]. To this purpose, both meristic traits (for distinguishing taxa by means of classification trees) and external morphology (investigated using geometric morphometrics) were analysed. In addition, morphological identification was verified on a subsample of specimens based on two nuclear regions, the Cyfun P (Cyprinid formerly unknown nuclear Polymorphism), a non-coding region of unknown function [39], and the Recombination Activating Gene 1 (RAG-1) and on the mitochondrial cytochrome b (cyt *b*) gene.

These different datasets were later combined in a quantitative statistical model of trait covariation.

## Materials and Methods

### Sampling

A total of 145 specimens of *S. lucumonis*, *S. squalus*, *T. muticellus* and *R. rubilio* were collected from 9 sampling sites within the lower section of the Tiber River basin (Table 1) using electrofishing procedures. Specimens were anesthetised with a 0.035% MS 222 (Tricaine Methanesulfonate) solution. The left lateral view of each specimen was photographed in the field for shape and meristic count analyses. A small portion of the pelvic fin was removed and fixed in 90% ethanol for genetic analysis. The procedures used for fish sampling were carried out in agreement with relevant legislation (CEN EN 14011/2003 - Water quality - Sampling of fish with electricity), avoided animal sacrifice and allowed for the live release of sampled specimens after data collection. Fish sampling were authorized by the Dipartimento Istituzionale e Territorio of the Regione Lazio (Prot. n. 526425). Specimens were initially identified according to external morphology using Bianco & Ketmaier [30] as reference for *S. lucumonis* and Gandolfi et al. [34] for the remaining three taxa. Taxonomic classification was further conducted in accordance with Kottelat & Freyhof [31]. Meristic counts and shape analyses through geometric morphometrics (GM) were carried out for the entire sample, whereas genetic analyses (based on nuclear and mitochondrial markers) were performed on a sub-sample.

**Table 1:** Details on the sampling sites in Tiber River basin and on number of individuals collected. Sl stands for *Squalius lucumonis*, Ss for *Squalius squalus*, Tm for *Telestes muticellus*, Rr for *Rutilus rubilio*, respectively.

GPS Position – WGS1984	LocationCode	N			
		Sl	Ss	Tm	Rr
42°09′ 10.71″ N –12° 38′ 47.33″ E	Pc	10	10	−	4
42° 09′ 58.00″ N –12° 43′ 27.00″ E	Sp	10	9	6	2
42° 09′ 07.40″ N –12° 41′ 28.70″ E	Fc	3	−	−	−
42° 00′ 32.20″ N –12° 28′ 33.70″ E	Pp	5	−	−	−
42° 08′ 00.00″ N –12° 39′ 54.10″ E	Ro	6	−	−	−
42° 05′ 00.70″ N –12° 41′ 48.90″ E	Ff	6	−	−	−
42° 10′ 22.80″ N –12° 32′ 41.20″ E	Ra	10	−	9	14
41° 55′ 54.00″ N –12° 43′ 55.10″ E	Fp	5	6	2	2
41° 55′ 19.71″ N –12° 46′ 03.54″ E	Sv	10	1	10	5
**Total**		**65**	**26**	**27**	**27**

### Analysis of Meristic Counts

Meristic characters are countable structures occurring in series (e.g. myomeres, vertebrae, fin rays). Six meristic characters were inspected in this study (Table S1), namely the number of scales of the lateral line (NSLL), above the lateral line (NSALL), under the lateral line (NSULL), and the number of rays of the dorsal fin (NRDF), left pectoral fin (NRPF), and anal fin (NRAF). A classification and regression tree (CRT) [40] was trained to build a species identification key based on meristic characters. This method has only recently been recognised as a useful tool for classification analysis by ecologists [41]. In the present study, all CRT procedures were performed using the software SPSS Statistics v17.0 (SPSS, Inc., 2009, Chicago, IL, www.spss.com). The six meristic counts were used as ordinal predictors of species, which was the dependent variable.

### Shape Analysis

**Figure 1:**
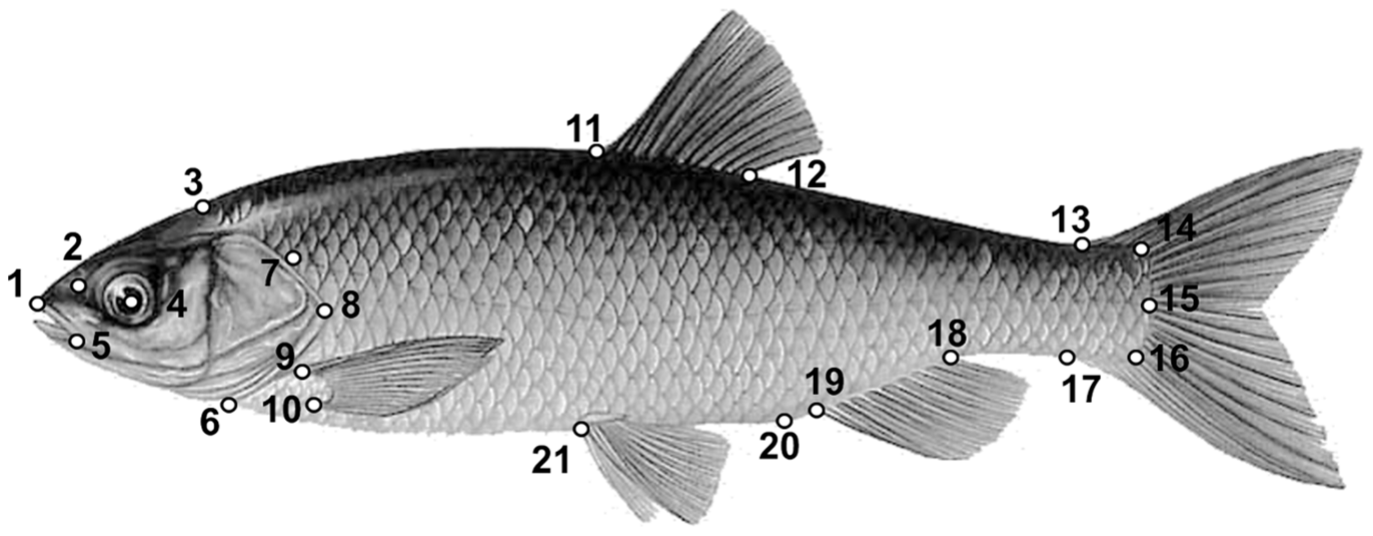
Landmarks used for Geometric morphometrics analysis of external shape. (1) Snout tip; (2) nostril; (3) beginning of scales coverage on the dorsal outline; (4) centre of the eye; (5) posterior extremity of the premaxillar; (6) insertion of the operculum on the ventral lateral profile; (7) beginning of the lateral line; (8) posterior extremity of the operculum; (9) superior and (10) inferior insertions of the pectoral fin; (11) anterior and (12) posterior insertions of the dorsal fin; (13) superior and (17) inferior insertions of the caudal fin; (14) superior and (16) inferior insertion of the caudal peduncle: (15) posterior body extremity; (18) posterior and (19) anterior insertion of the anal fin; (20) anus; (21) anterior insertion of the pelvic fin.

Each fish was measured (standard length, SL), and twenty-one landmarks (Fig. 1) were recorded on each digital image using the software TPSDIG [42].

Configurations were scaled to unit centroid size, translated to a common centroid, and rotated to minimise the least squared residuals between corresponding landmarks [43]. Residuals from the registration were analysed with the thin-plate spline (TPS) interpolating function [44]. A canonical variate analysis (CVA) was performed on the W’ matrix of partial warps [45], [46] using MorphoJ [47]. CVA is often used to identify the shape features that best distinguish multiple groups of specimens by determining the linear combinations of the original variables that display the greatest variance between groups relative to the variance within groups. Group membership is assumed to be known *a priori*. The emphasis is on the degree of separation between groups and on the probability of correct and incorrect classification of each observation. The robustness of assignment was assessed through a leave-one-out resampling cross-validation procedure. Splines relative to the extreme positions of the canonical axes were obtained using TPSREGR [48]. Differences between shapes were visualised using deformation grids [44], which represent the most robust visualisation tool for synthesising the entire pattern of shape variation. In addition, the CVA procedure in MorphoJ carries out a leave-one-out cross-validation method (number of permutation runs = 1000) to assess the reliability of classifications.

### Genetic Analyses

The DNA of 28 specimens (Table S2) (primarily from *S. lucumonis* and randomly chosen within each species) was extracted from ethanol-preserved fin tissue following the salt extraction protocol of Aljanabi & Martinez [49]. Amplifications of the three selected markers were performed using primers and protocols available in the literature [50]–[52] and reported in Table S3.

The Cyfun P band length (310–440 base pair, bp) was used for a rough species attribution and to verify the occurrence of nuclear hybrid genomes. This region is indeed known to show intergeneric diagnostic length and/or sequence polymorphism in leuciscine fishes [39]. Amplicons were also purified with SureClean (Bioline) and sequenced with an automated DNA sequencer (Macrogen Inc.). NCBI’s BLAST software was used for similarity searching of obtained sequences, that were then deposited in the GenBank database (Accession Numbers: JQ286163–JQ286167, JQ286169), aligned with ClustalX [53] and subsequently manually adjusted. Sequences allowed a more accurate species attribution.

The nuclear RAG1 (840 bp) and the mitochondrial cyt *b* (1131 bp) amplicons were processed as previously described (Accession Numbers: JQ286150–JQ286162 and JQ799135–JQ799137 for cyt b, and KC478779–KC478783 for RAG1) and used in subsequent phylogenetic analyses. Cyfun P sequences were excluded from these analyses as the presence of the numerous indels causes ambiguity in the alignment. In phylogenetic reconstructions,sequences of the four species retrieved from GenBank were also added (Table S4); for cyt *b*, only sequences longer than 1100 bp were considered. *Pseudorasbora parva* was used as outgroup, since sequences of both markers, from the same individual, were available. Phylogenetic analyses were performed independently on each gene and then on the combined dataset, using both maximum-likelihood (ML) and Bayesian inference (BI) analysis. Modeltest 3.7 [54] and Mrmodeltest 2.3 [55] were used to select the evolutionary model that best fits the data set for each data set for the ML and the BI analyses, respectively. The Akaike’s information criterion (AIC) [56] was used.

ML tree reconstructions were conducted in PhyML v2.4.4 [57], using heuristic search with the NNI swap algorithm and 1000 bootstrap replicates. Bayesian analyses were implemented in MrBayes v3.1.2 [58] performing two independent runs of four Markov chains each for 1,000,000 generations. The average standard deviation of split frequencies was close to 0.003 when the runs were finished. Trees were sampled every 1,000 generations, burn-in trees (25%) were discarded and a 50% majority-rule consensus tree was estimated.

Species tree reconstruction was performed using the coalescent-based Bayesian species tree inference method with the ∗BEAST (Bayesian Inference of Species Trees from Multilocus Data) protocol [59] implemented in the software BEAST v1.7.4 [60]. The input file was properly formatted with the BEAUti utility included in the software package, using the same partition scheme of the concatenated analysis. Two runs, each of 50×10^6^ generations (samplefreq = 5000 and 10% burnin), were conducted. Tracer v1.5 [61] was used to check for convergence and normal distribution. The treefiles were combined in Logcombiner v1.7.4 and maximum clade credibility trees were created with TreeAnnotator v1.7.4, both programs are implemented in BEAST v1.7.4. Finally, a species tree was inferred and viewed using FigTree v1.4.0 [62].

We used the posterior predictive checking approach of Joly et al. [63], implemented in JML v1.0.1 [64] to test whether the incongruence between nuclear and mitochondrial genotypes was the result of incomplete lineage sorting or hybridization. We used both the RAG1 and cyt *b* datasets and the parameters yielded by Modeltest in JML running. The software calculates minimum pairwise sequence distances between the simulated sequences of individuals including all possible comparisons, and tests for each dataset whether the observed minimum distance between sequences of two species is smaller than those expected under the simulated model that does not account for hybridization. Gene trees and sequences were simulated separately for the mitochondrial (mt) and the nuclear (n) datasets using the relative substitution rates adopted in BEAST. Appropriate heredity scalars were selected for simulations of nuclear (2) and mitochondrial (0.5) markers, respectively. A significance level of 0.1 was specified in all runs.

Genetic distances obtained for the 28 RAG1 and cyt *b* sequences belonging to the four species (Table S5) were used as input for a principal coordinate analysis (PCOA) performed with the PAST program [65]. The scores of the specimens on these axes were used as variables in the morphology and genetics comparative analyses.

### Integration between Morphology and Genetics: Partial Least-square Analysis

A partial least-square (PLS) analysis [42], [66] was conducted to explore the pattern of covariation between shape and genetic distance for the four species. The PLS analysis is a multivariate correlation technique that identifies pairs of linear combinations between two sets of input variables (here, shape and the two axes of the PCOA performed on the matrix of genetic distances). The PLS analysis produces ordered pairs of ‘latent’ vectors, one per each input dataset, that account for the maximum amount of information covariation between the two original sets of variables [66]–[68]. The amount of covariance explained by a pair of latent vectors (represented by the correlation coefficient ‘R’) and its ‘strength’ (estimated by permutations, TPSPLS [69]) provide a statistical assessment of the association between the two datasets. R can serve as a measure of the integration of shape and sets of other variables [43], [66]. This PLS approach has been successfully used to compare shape between different body regions [70] or to relate shape to other aspects of fish ecology, such as diet [71], [72]. However, this study represents the first attempt to use PLS analysis to relate shape and genetic data. For this analysis, we used as input (1) the landmarks data and (2) the scores on the PCOA axes for each specimen, which summarise the pattern arising from genetic analysis.

## Results

### Analysis of Meristic Counts

**Figure 2:**
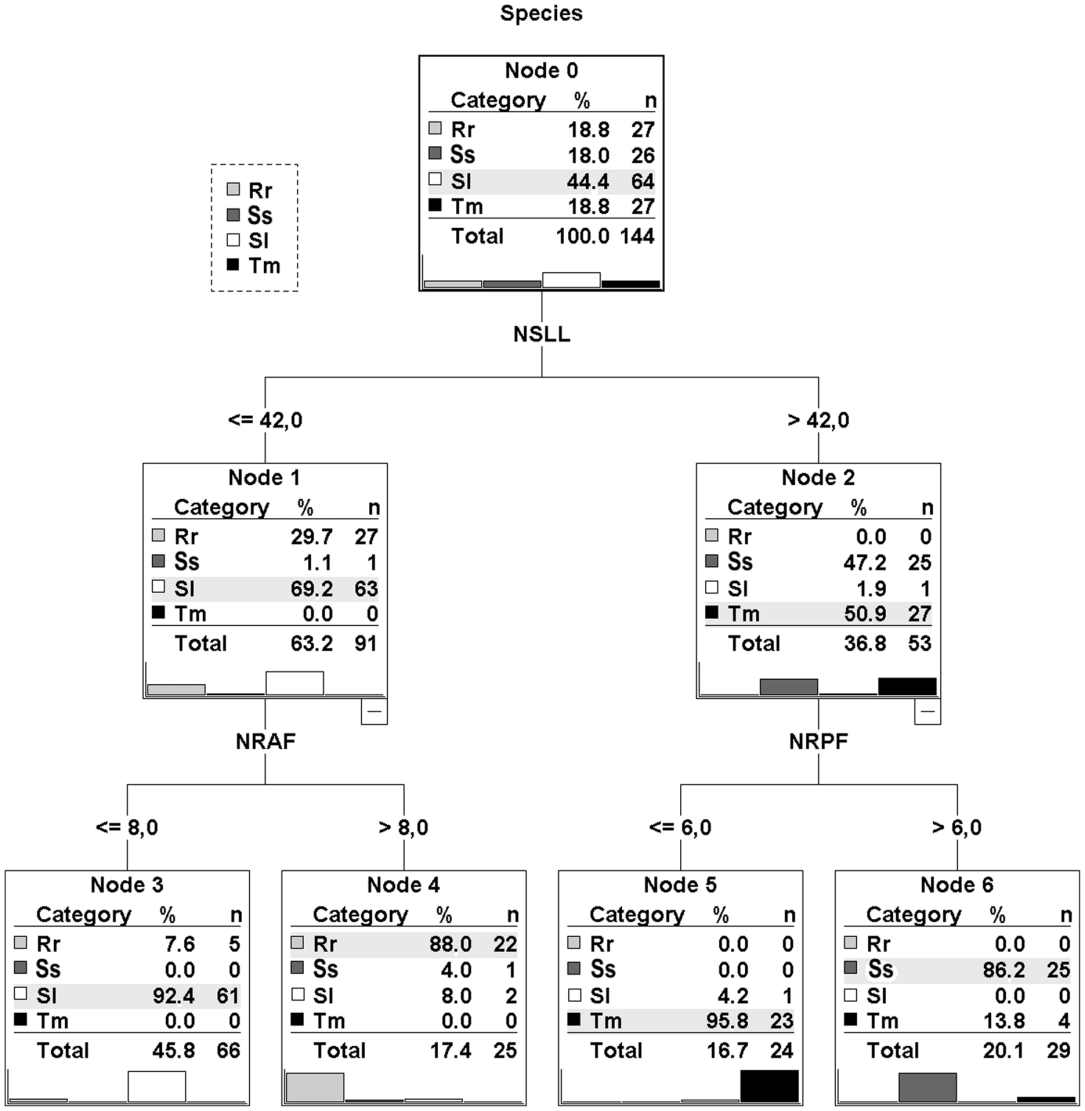
Classification tree obtained from the CRT analysis. NSLL: number of scales of the lateral line; NRAF : number of rays of the anal fin; NRPF : number of rays of the left pectoral fin. Rr: *R. rubilio*; Ss: *S. squalus*; Sl: *S. lucumonis*; Tm: *T. muticellus*.

The CRT analysis produced a tree with four terminal nodes separating all four species (Fig. 2). The combination of NSLL, NRAF and NRPF characters allowed the discrimination among species The cross-validation procedure reported an overall correct classification percentage of 91%, ranging within species from 81.5% (*R. rubilio*) to 96.2% (*S. squalus*).

When misclassified specimens are excluded from analysis, some subtle differences can be detected in the ranges of the values of meristic characters for the four species (Table S1).

### Shape Analysis

**Figure 3:**
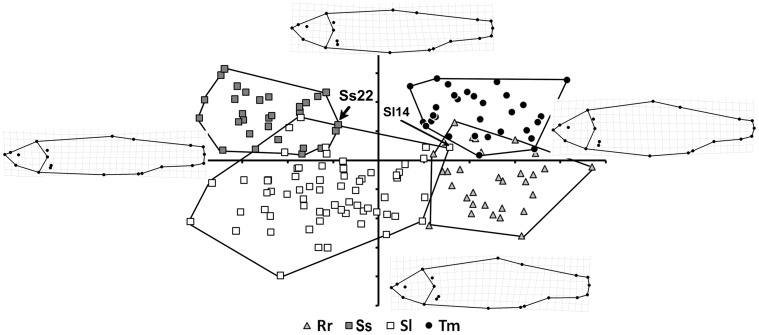
CVA output of the overall comparisons of the four taxa; the pattern described by the first two discriminant axes is shown. Convex hulls are used to delimitate groupings; splines illustrate shape changes along each axis. Rr: *R. rubilio*; Ss: *S. squalus*; Sl: *S. lucumonis*; Tm: *T. muticellus*. See text for arrowed individuals.

The results of the CVA were highly statistically significant for comparisons between the four species: the distribution along the two discriminant axes shows that intra-specific shape differences are much lower than the inter-specific ones (Procrustes distance = 0.038, Mahalanobis distance = 14.7161, p<0.01). Thus, the plot in Fig. 3 tends to segregate each taxon into a different quadrant, generating four, largely non-overlapping, groups of specimens. The magnitude of intra-group variation is largely consistent and comparable among three of the four species, whereas it is twice as large in *S. lucumonis*. The shape changes underpinning each axis can be examined by looking at the splines at the extremes of the axes. CVA1 describes a progressive increase in the lateral profile, which passes from a streamlined condition to a more discoidal one. This change mainly involves the head (see landmarks 2, 3 and 6) and trunk (see landmarks 11, 12, and 21) regions, whereas the tail region remains largely unchanged. The pattern depicted by this axis discriminates the two *Squalius* species from the *R. rubilio* and *T. muticellus* pair. In contrast, CVA2 describes a substantial change in the dorsal profile, which becomes more flattened in the transition from negative coordinates to positive ones along this axis. This shape change is captured by landmarks 3, 11, and 12. The dorsal profile of the head is also involved in this modification, such that the relative position of the eye (landmark 4) moves to a higher position. In addition, CVA2 describes a shortening of the height of the caudal peduncle (landmarks 13 and 17). All shape changes captured by this axis are crucial to distinguish between *S. squalus* and *S. lucumonis* and between *R. rubilio* and *T. muticellus.*

The results of the cross-validation test are shown in Table 2. The model is efficient overall in identifying species (93.4% of cases). The highest rate of misclassification occurred in comparisons of *R. rubilio* with *T. muticellus*, whereas the lowest one occurred for the comparison between *R. rubilio* and *S. squalus*. In addition, the number of misclassified specimens differed from that arising from the observation of CVA pattern (Fig. 3). This difference reflects the fact that only the first two discriminant axes are visualised in Fig. 3, whereas the cross-validation test was carried out using all of the shape data.

**Table 2:** Results of the cross-validation test performed on shape data. Sl: *S. lucumonis*, Ss: *S. squalus*, Tm: *T. muticellus* and Rr: *R. rubilio*.

Groups	N	Allocated to		Percent missclassified		
		**Sl**	**Ss**			
Sl	65	62	3		4.6	
Ss	26	4	22		15.3	
		**Sl**	**Tm**			
Sl	65	63	2		3.1	
Tm	27	1	26		3.7	
		**Sl**	**Rr**			
Sl	65	62	3		4.6	
Rr	27	2	25		7.4	
		**Ss**	**Tm**			
Ss	26	23	3		11.5	
Tm	27	3	24		11.1	
		**Ss**	**Rr**			
Ss	26	26	0		0	
Rr	27	0	27		0	
		**Tm**	**Rr**			
Tm	27	25	2		7.4	
Rr	27	4	23		14.8	

In the pairwise comparisons (Fig. S1), the landmarks that best captured shape differences between taxa are those of the dorsal (11–12), pelvic (21) and anal (18–19) fins and the anus (20). However, the landmarks for the pectoral fin (9–10), caudal fin (13–17), and eye position (4) have a non-negligible role in some comparisons. Looking at Figs. 3 and S1, it seems that *S. lucumonis* differs from the other taxa in the position of insertion of the dorsal fin (which is shifted in the caudal direction if compared to *T. muticellus*) and by its high lateral profile relative to *R. rubilio* and *S. squalus*.

### Genetic Analyses

The presence of a single Cyfun P band was detected for each individual after amplicon size analysis on agarose gel. As expected [39], Cyfun P showed a larger variability at intergeneric (*Squalius*, *Rutilus* and *Telestes*) than at intrageneric (*Squalius*) level. Bands obtained ranged from approximately 310 bp in *T. muticellus* to 440 bp in *R. rubilio. S. lucumonis* and *S. squalus* showed similar intermediate band length (approximately 410 bp). Six different haplotypes were identified among the 28 analysed individuals, two typical of *S. lucumonis* (99.7% similarity), one of *S. squalus,* one of *T. muticellus* and two of *R. rubilio* (98.5% similarity). In spite of band length results, the alignment of the two *Squalius* species allowed to distinguish them by three diagnostic substitutions (two transversions and one transition) and by the presence of a 17 bp deletion at the 3′ end in *S. lucumonis*. Two individuals, one morphologically identified as *S. squalus* (Ss22) and one as *S. lucumonis* (Sl14), showed a *S. lucumonis*-like and a *R. rubilio*-like nuclear sequence, respectively. Sequence BLAST provided 99–100% similarity with GenBank specimens assigned to the same species, with the exception of *S. lucumonis,* whose sequences are not available in the database. In these case 99% similarity was obtained with the Balkan *S. tenellus, S. microlepis* and *S. illyricus.* When our sequences were aligned with those of other *Squalius* species present in Genbank (*S. aphipsi, S. cephalus, S. illyricus, S. microlepis, S. squalus, S. tenellus, S. zrmanjae*), the *S. lucumonis* 17 bp indel was shared only by the three above mentioned Balkan species.

Out of the 840 bp sequenced for RAG1 gene, 24 variable sites (2.8%), corresponding to 5 haplotypes, were identified. Sequence similarity within species ranged from 98.2% in *S. squalus,* to 100% in *T. muticellus* and *R. rubilio*. Higher percentages were detected for *S. lucumonis* and *S. squalus*, (99.9% and 100%, respectively), when the specimen Sl14 and Ss22 were excluded from the analysis. Indeed, RAG1 confirmed CyfunP results on these individuals (see above). Sequence similarity between species ranged from 98.1% (between *S. lucumonis* and *S. squalus*) to 98.8% (between *R. rubilio* and *T. muticellus*). BLAST search in GenBank did not provide informative data, as 99% similarity was obtained with many different leuciscine species, due to the general small substitution rate of nuclear genes and to the tendency of the program to round up the percentage of identity of the match to the nearest whole number. However when BLAST alignments were considered, the smaller number of substitutions for each species (1 for *T. muticellus*, 3 for *S. lucumonis*, 2–4 for *S. squalus*) was obtained with conspecific sequences, when available. The RAG1 phylogenetic trees obtained by the ML and BI analyses showed similar topologies (Fig. S2). The four species clustered in well-separated, well-supported clades, each of which included the conspecific sequences retrieved from GenBank.

Out of the 1131 bp sequenced for cyt *b* gene 248 variable sites (21.9%), corresponding to 16 haplotypes, were identified. Sequence similarity within species ranged from 87.1% (*S. lucumonis*) to 99.6% (*S. squalus* and *R. rubilio*). A higher percentage (99.1%) was detected for *S. lucumonis* when the specimen Sl14 was excluded from analysis. Indeed, this individual, according to nuclear marker data, showed a *R. rubilio*-like cyt *b* sequence. Sequence similarity between species ranged from 85.5% (between *R. rubilio* and *T. muticellus*) to 92.9% (between *S. lucumonis* and *S. squalus*). BLAST search of our sequences in GenBank, showed the highest percentage of similarity (99%) with specimens assigned to the same species. *S. squalus* sequences, in addition to exhibiting similarities to haplotypes of samples deposited with either this species name [73], [74] or the former valid one [2], [75], also showed 99% similarity with other species (*S. lucumonis* and *S. zrmanjae*) as already reported by Durand et al. [2].

**Figure 4:**
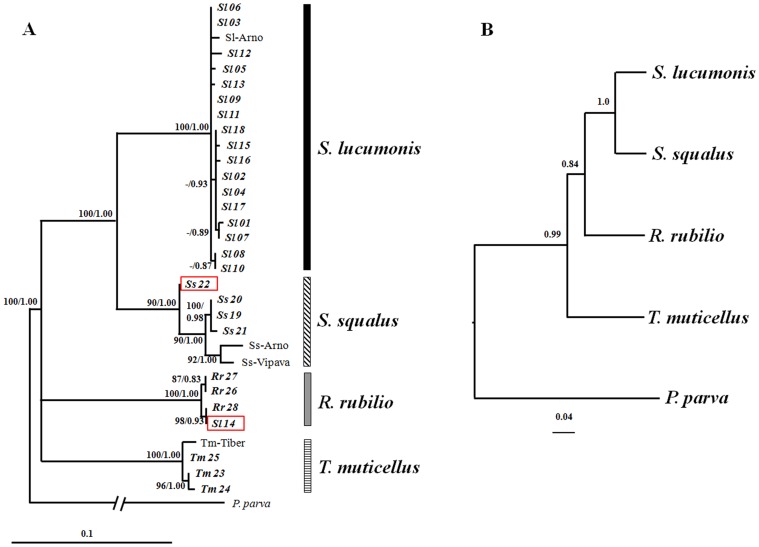
Species trees estimated with concatenation and coalescent approaches. Panel A: ML and BI trees of concatenated dataset (RAG1 and cyt *b* sequences); bootstrap values (>70%) and Bayesian posterior probabilities (>0.7) are reported. Sl-Arno, Ss-Arno, Ss-Vipava, Tm-Tiber idicate specimens whose cyt *b* and RAG1 sequences were available in GenBank (see Table S4). Specimen Ss22 showing a *S. lucumonis*-like nuclear sequence, and Sl14 showing both mitochondrial and nuclear *R. rubilio*-like sequences, are boxed. Panel B: Phylogeny estimated with *BEAST; posterior probabilities shown for all nodes.

The phylogenetic trees obtained using separately nuclear (Fig. S2) and mitochondrial (Fig. S3) markers support the presence of four clades, each including conspecific sequences retrieved from GenBank. However their topologies show some incongruences in the branching of the four lineages. Two of the cyt *b* sequences retrieved from GenBank (AJ252818 and AJ252819) corresponding to specimens classified (based on morphology) as *S. lucumonis* but having *S. squalus*-like cyt *b* (Tib 1 and Tib 2) haplotypes [2] clustered with those of *S. squalus.* The topology of combined data trees (Fig. 4a) was identical to that obtained by cytochrome b, due to the higher number of variable sites of this gene compared to RAG1. The species tree (Fig. 4b) confirms the results obtained in gene genealogies supporting the distinction of four groups that correspond to the four species.

The analysis performed in JML showed consistent results for the nDNA and for the mtDNA markers, as minimum genetic distances observed between *S. lucumonis* and *S. squalus* were all non-significant smaller than simulated values. This indicate that incomplete lineage sorting alone can explain our data.

### Integrating Morphology and Genetics: Partial Least-square Analysis

The complete suite of morphological (CRT and GM) and molecular (nuclear and mitochondrial) analyses was carried out for the 28 individuals (Table S2). In 21 specimens (75%), there was complete concordance of data. In the remaining 25% of cases, however, species assignment was equivocal, with disagreement between and within morphological (CART and Shape) and/or molecular (mitochondrial and nuclear) data. For example, the specimen Sl14 was assigned to *S. lucumonis* according to morphological data and to *R. rubilio* according to molecular data. In analyses of morphological data, five individuals (Table S2) were differently assigned by the CRT and Shape analyses. In two cases (Sl16 and Sl02) molecular data confirmed the CRT assignment, and in three cases (Rr26, Sl03, Tm23) molecular data confirmed the shape assignment. Nuclear and mitochondrial markers provided completely concordant data, with the exception of the already mentioned Ss22 individual that was initially classified as *S. squalus* according to meristic and morphometric traits.

**Figure 5:**
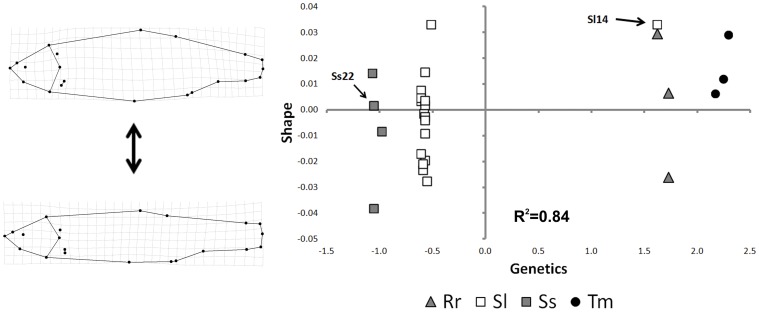
Scatterplot representing the pattern obtained by partial least square (PLS) analysis of shape (GM) and genetic (PCOA axes) data. Splines show shapes relative to the extremes of the y-axes. Specimen Ss22 and Sl14 are indicated by arrows.

The PLS analysis detected a significant (p<0.05) monotonic relationship between shape and genetic distance for the four taxa (Fig. 5). When approximated by a linear relationship, it returned an R^2^ of 0.84. The two species of genus *Squalius* are close to each other in the negative half plane of latent vector 1, whereas *R. rubilio* and *T. muticellus* are located in the positive half plane. While the four species evidenced comparable levels of morphological and genetic variability, the taxa are mainly separated along the latent vector 1 (genetic information). This suggests that the degree of genetic distance between taxa is larger than those of morphological distance. Specimen Sl14, misclassified by the shape analysis, was the only specimen falling on the positive semi-plane of this axis.

## Discussion

Our results, although based on specimens collected only in the Tiber River, and thus not representative of the entire distribution area, indicate that *S. lucumonis*, *S. squalus*, *T. muticellus* and *R. rubilio* are four valid and differentiated taxa and unambiguously confirm the status of *S. lucumonis* as a valid species.

The analyses of meristic counts and external shape reveal that the four species are characterised by significantly different, non-overlapping external morphologies. The specimens located in the regions of CVA overlap (Fig. 3), some of which were misclassified by the cross-validation procedure (Table 2), reflect a limitation in the efficiency of the model. The classification tree built on the basis of meristic characters separated the four taxa primarily by their NSLL and NRPF values; a third character (NRAF) appears to be important for distinguishing *S. lucumonis* from *R. rubilio*. Moreover, the two morphological analyses, each independently, separated the four taxa. In the CVA, a higher-order distinction was established between the genus *Squalius* (located at negative scores of axis CV1) and the two other species (*R. rubulio* and *T. muticellus*) (located at positive scores), suggesting some degree of intra-genus similarity. In contrast, the CRT analysis most strongly discriminated between pairs that did not reflect taxonomic relationships. However, the two critical meristic count characters identified by CRT analysis appear to be related to the shape changes identified by geometric morphometric analysis, as NSLL is a function of the absolute length of the body profile (being larger for a streamlined shape and shorter for a discoidal one) and both NRPF and NRAF are functions of the variation in the position of landmarks describing the insertions of these fins. For example, NRAF variation corresponds to one of the major external shape changes detected by geometric morphometrics in the pairwise comparison between *R. rubilio* and *S. lucumonis* (Fig. S1). Overall, the results of the morphological analyses reinforced one another, and the analyses provided different quantitative tools for distinguish among them.

Nuclear marker did not identify the presence of hybrid nuclear genomes either by amplicon band length analysis on agarose gel (Cyfun P) or by sequencing (Cyfun P and RAG1). However, the additional evaluation of the cyt *b* sequences allowed the identification of one individual (Ss22) with mixed genetic features, i.e., with a *S. squalus* cyt *b* haplotype and *S. lucumonis* nDNA. Consequently this individual shows an incongruent clustering in the two gene trees. Inconsistencies between gene trees, could be caused either by introgressive hybridization (ancient or recent) or incomplete lineage sorting [76], that can both generate very similar phylogenetic patterns [63]. Based on cyt *b* analysis, Durand et al. [2] already suggested incomplete lineage sorting between *S. lucumonis* and *S. squalus.* Indeed these authors identified three cytochrome b haplotypes from 9 brook chub individuals from Tiber Rivers, arranged into two different clades. One haplotype, shared by 6 specimens, formed the *S. lucumonis* clade, while the remaining two haplotypes (Tib1 and Tib2, 3 specimens) were included within the formerly “Adriatic lineage” of *S. cephalus,* now *S. squalus*. Neverthless, also introgressive hybridisation has been commonly reported in *Squalius,* often involving *S. cephalus* lineage [77]–[79]. Distinguishing between these two alternatives is not easy, and different methods were developed at this scope [63], [80], [81], [82]. The tests performed in JML suggests that the observed pattern is due to incomplete lineage sorting, a phenomenon that occurs when an ancestral species undergoes several speciation events in a short period of time. In this case the ancestral gene polymorphism is not fully resolved into two monophyletic lineages. However as “the difference in minimum distances between species simulated under incomplete lineage sorting and hybridization scenarios is not as distinct when short sequences are considered” [63], further and focused investigations are necessary to definitively solve this critical issue in every aspect.

Finally, the application of PLS analysis reconciles the results of the morphological (GM) and genetic analyses. The PLS analysis demonstrates that the information provided by these two different approaches is largely consistent and that their combined use can resolve cases of misclassification.

We detected a monotonic, progressive trend from *R. rutilus* and *T. muticellus* (the species characterised by a more discoidal body shape and with high scores on Shape axis) to the *Squalius* species (characterised by a more streamlined body shape and with low scores on Shape axis) (Fig. 5). *S. lucumonis*, however, spanned to the larger positive values on Shape axis, showing the largest shape variation among the four taxa. In this context, the misclassification of specimen Sl14 by the shape analysis can be explained by the scores at the extreme of the range that comprise *S. lucumonis* specimens along the second axis. Future studies could investigate if this pattern is related to ecomorphological factors (e.g., an adaptation to diverse ecological lotic systems, such as brooks, creeks, or small streams).

### Conclusions

The morphological and genetic analyses reported here indicate that an integrated approach can be useful in resolving taxonomic relationships and in conservation management. This is particularly true for fish groups such as the Leuciscidae, which are characterised by large morphological intraspecific variation, interspecific overlap of morphological characters and frequent hybridisation events. Gilles et al. [7] reported that, in some Leuciscidae genera, the inferred number of species can vary depending on whether morphological or molecular criteria are adopted and that a comprehensive interpretation is possible only if a combination of approaches is applied. Our data provide evidence of morphological variation in the four taxa examined, with approximately 5 of 28 individuals ascribed to different taxa on the basis of CRT and shape characters. In contrast, if individuals with “hybrid” genotypes are present, they are undetectable in the population. Therefore, not only are morphological and genetic markers necessary for the correct assignment of individuals, both nuclear and mitochondrial datasets are also required for reliable genetic analyses. Our approach contributes to a broader knowledge of Italian leuciscines and provides robust elements for revisiting national and regional conservation strategies.

The presence in our dataset of an individual with mixed genetic traits (mtDNA of one *Squalius* species and nDNA of the other) poses a question on a strict application of the phylogenetic species concept to the two, otherwise monophyletic, groups (i.e. *S. lucumonis* and *S. squalus*). However Chambers [83], considering that speciation is a gradual process and that does not always exist a single perfectly-fitting species concept, suggests a synthetic model that allows the classification of pair of taxa applying a two step decision matrix. In the context of this synthetic model no further doubt on the status of *S. lucumonis,* at least in the Tiber River, is reasonable. This evidence, in light of the recent reassessment of this taxon into the “Endangered” IUCN category, highlights the need for immediate conservation actions. These actions require the inclusion of this species in the lists of fish species of conservational interest (*sensu* Habitat Directive), including the lists of the Latium Region [84], where a large number of populations are located. In addition conservation measures also need to address habitat restoration and specific legislation to protect residual and fragmented populations of this endemic species.

## Supporting Information

Figure S1 **Pairwise comparisons between the four taxa in which shape differences are stressed by a factor of 3.0X and visualised as vector arrows that start from the consensus configuration and end at the corresponding position of the taxon mean shape.**
(TIF)

Figure S2 **ML and BI trees based on RAG1 gene sequences using the TVMef+I G model of nucleotide substitution.** Bootstrap values (>70%) and Bayesian posterior probabilities (>0.7) are reported. Specimen Ss22 and Sl14, showing a *S. lucumonis*-like and a *R. rubilio*-like nuclear sequence, respectively, are boxed.
(TIF)

Figure S3 **ML and BI trees based on cyt** ***b*** **gene sequences using the GTR+I+G model of nucleotide substitution.** Bootstrap values (>70%) and Bayesian posterior probabilities (>0.7) are reported. Specimen Ss22 showing a *S. lucumonis*-like nuclear sequence, and Sl14 showing both mitochondrial and nuclear *R. rubilio*-like sequences, are boxed.
(TIF)

Table S1 **Meristic characters (MC) inspected and ranges of values observed.** In parenthesis values computed exclusively on specimens correctly classified by CRT.*marks meristic characters used for CRT.
(DOC)

Table S2 **Species assignment for each of the 28 individuals identified with both morphological (CART and Shape) and molecular (nuclear: Cyfun P and RAG1; mitochondrial: cyt** ***b*** **) approaches.** Sl stands for *Squalius lucumonis*, Ss for *Squalius squalus*, Tm for *Telestes muticellus*, Rr for *Rutilus rubilio*, respectively. In bold, individuals for which the species assignment was not univocal; in brackets Genbank accession numbers.
(DOC)

Table S3 **Primers and protocols used for amplifications.**
(DOC)

Table S4 **Pairwise genetic distances used in PCOA analysis.**
(DOC)

Table S5 **Available sequences for RAG 1, cyt** ***b*** **(GenBank Accession Number) and combined based phylogenetic analyses.**
(DOC)
