# Supplementary figures and images for: Testing Species Delimitations in Four Italian Sympatric Leuciscine Fishes in the Tiber River: A Combined Morphological and Molecular Approach
Source: PLoS One. 2013 Apr 2;8(4):e60392. doi: 10.1371/journal.pone.0060392 (PMC3614999; doi:10.1371/journal.pone.0060392)

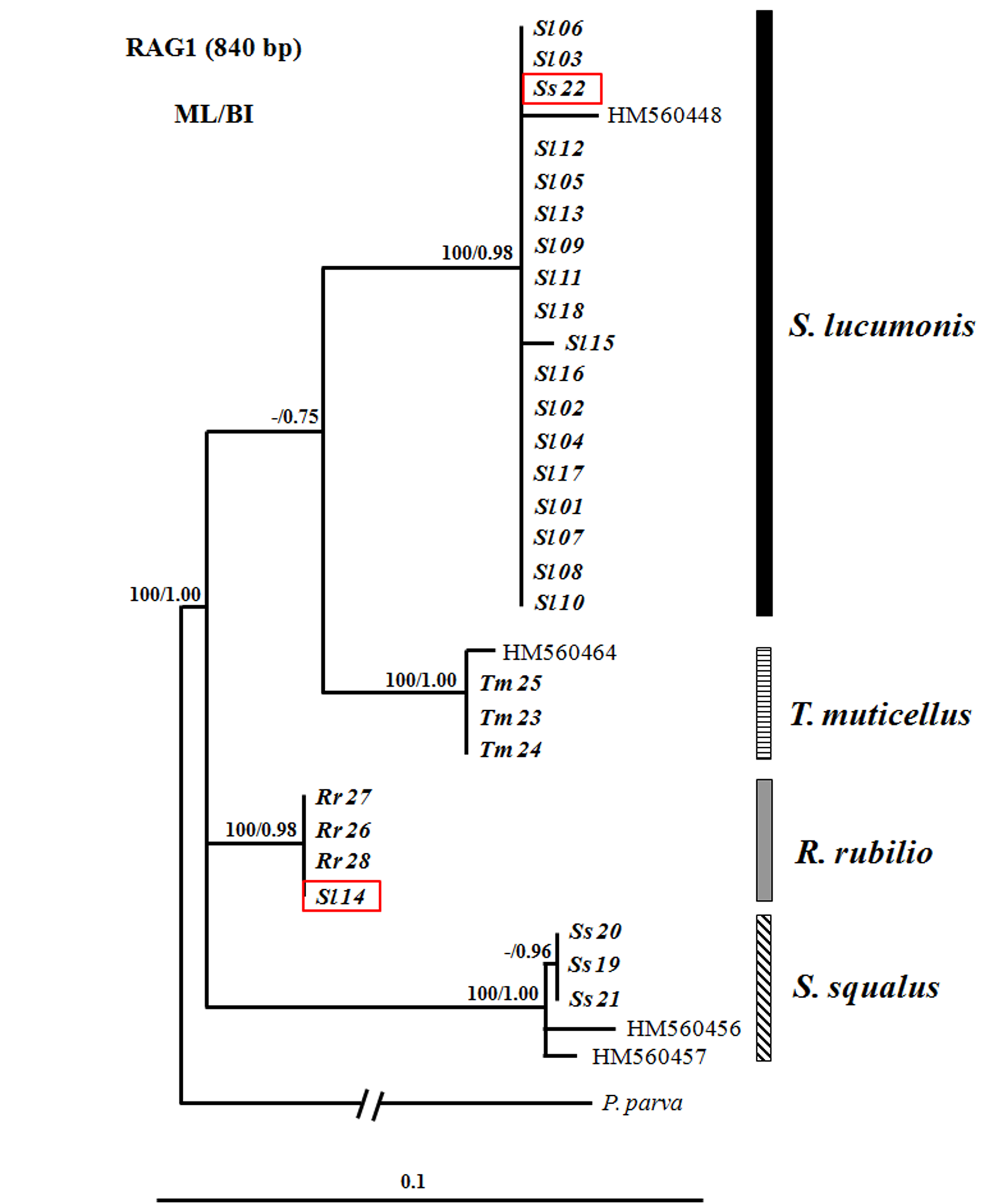

Supplement: Figure S2 — ML and BI trees based on RAG1 gene sequences using the TVMef+I G model of nucleotide substitution. Bootstrap values (>70%) and Bayesian posterior probabilities (>0.7) are reported. Specimen Ss22 and Sl14, showing a S. lucumonis-like and a R. rubilio-like nuclear sequence, respectively, are boxed. (TIF) [file pone.0060392.s002.tif]

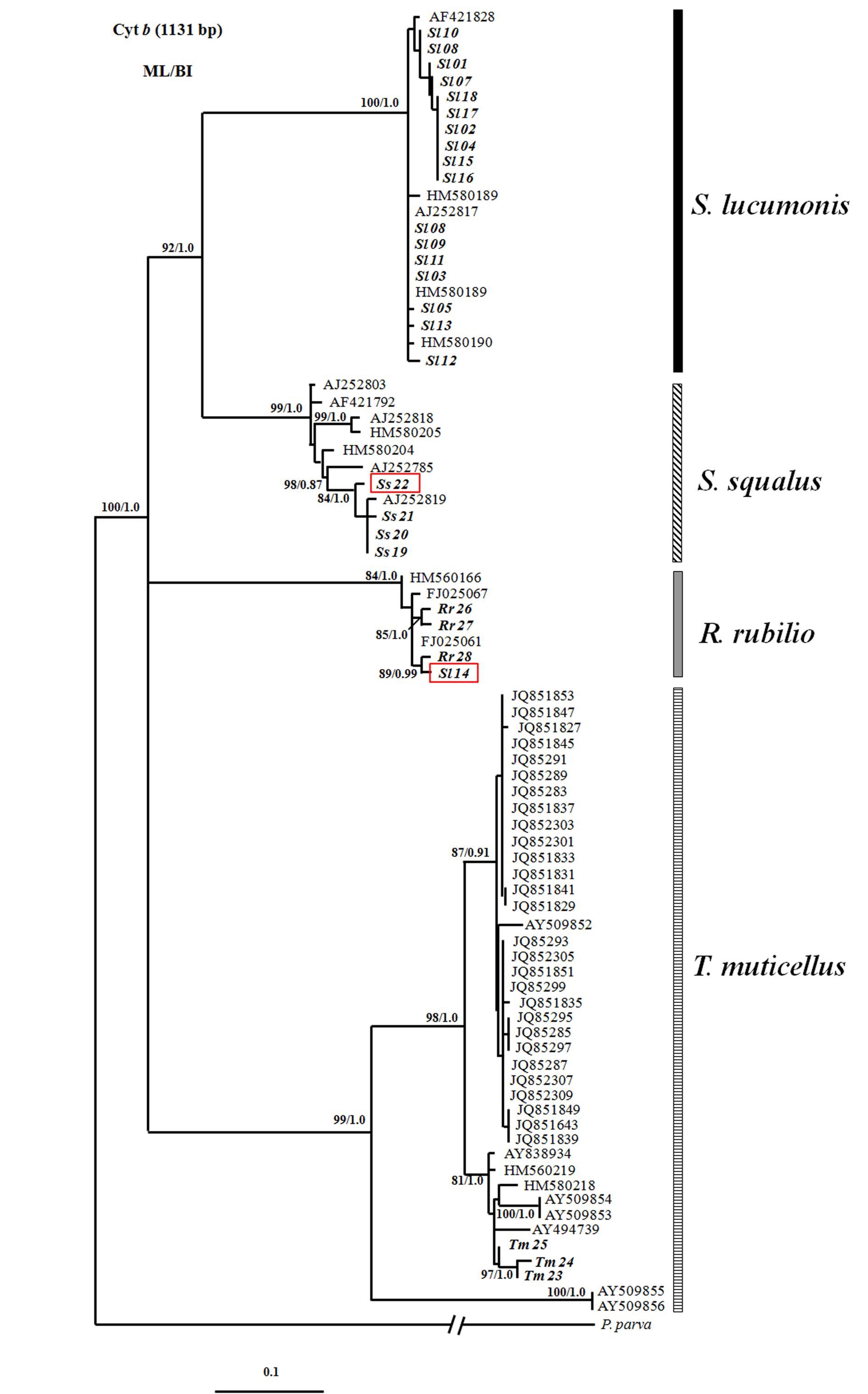

Supplement: Figure S3 — ML and BI trees based on cyt b gene sequences using the GTR+I+G model of nucleotide substitution. Bootstrap values (>70%) and Bayesian posterior probabilities (>0.7) are reported. Specimen Ss22 showing a S. lucumonis-like nuclear sequence, and Sl14 showing both mitochondrial and nuclear R. rubilio-like sequences, are boxed. (TIF) [file pone.0060392.s003.tif]
