# Supplementary material for: Testing Species Delimitations in Four Italian Sympatric Leuciscine Fishes in the Tiber River: A Combined Morphological and Molecular Approach
Source: PLoS One. 2013 Apr 2;8(4):e60392. doi: 10.1371/journal.pone.0060392 (PMC3614999; doi:10.1371/journal.pone.0060392)
Supplement: Table S1 — Meristic characters (MC) inspected and ranges of values observed. In parenthesis values computed exclusively on specimens correctly classified by CRT.*marks meristic characters used for CRT. (DOC) [file pone.0060392.s004.doc]

| **MC (code)** | **Observed ranges** | | | |
| --- | --- | --- | --- | --- |
|  | *S. lucumonis* | *S. squalus* | *T. muticellus* | *R. rubilio* |
| № of scales of the lateral line (NSLL)* | 37-43  (37-42) | 41-48  (43-48) | 45-52 | 34-40 |
| № of scales above the lateral line (NSALL)* | 7-9 | 7-8 | 7-10 | 7-9 |
| № of scales under the lateral line (NSULL)* | 3-4 | 3-4 | 4-5 | 3 |
| № of rays of the dorsal fin (NRDF)* | 7-8 | 7-9 | 7-9 | 8-9 |
| № of rays of the left pectoral fin (NRPF)* | 5-7 | 7 | 5-7 | 6-7 |
| № of rays of the anal fin (NRAF)* | 7-9  (7-8) | 8-9 | 7-9 | 8-9  (9) |
