# Supplementary material for: Testing Species Delimitations in Four Italian Sympatric Leuciscine Fishes in the Tiber River: A Combined Morphological and Molecular Approach
Source: PLoS One. 2013 Apr 2;8(4):e60392. doi: 10.1371/journal.pone.0060392 (PMC3614999; doi:10.1371/journal.pone.0060392)
Supplement: Table S2 — Species assignment for each of the 28 individuals identified with both morphological (CART and Shape) and molecular (nuclear: Cyfun P and RAG1; mitochondrial: cyt b ) approaches. Sl stands for Squalius lucumonis, Ss for Squalius squalus, Tm for Telestes muticellus, Rr for Rutilus rubilio, respectively. In bold, individuals for which the species assignment was not univocal; in brackets Genbank accession numbers. (DOC) [file pone.0060392.s005.doc]

| Species | Location  Code | Sample | Morphological | | |  | Molecular | | | |
| --- | --- | --- | --- | --- | --- | --- | --- | --- | --- | --- |
|  |  |  | CART | | Shape |  | Cyfun P | RAG1 | cyt *b* |  |
| *S. lucumonis* | Sp | Sl 01 | Sl | Sl | |  | Sl  (JQ286169) | Sl  (KC478779) | Sl  (JQ799136) |  |
|  |  | Sl 02 | Sl | **Ss** | |  | Sl  (JQ286169) | Sl  (KC478779) | Sl  (JQ286157) |  |
|  |  | Sl 03 | **Rr** | Sl | |  | Sl  (JQ286169) | Sl  (KC478779) | Sl  (JQ286162) |  |
|  | Fc | Sl 04 | Sl | Sl | |  | Sl  (JQ286169) | Sl  (KC478779) | Sl  (JQ286157) |  |
|  | Pp | Sl 05 | Sl | Sl | |  | Sl  (JQ286169) | Sl  (KC478779) | Sl  (JQ286161) |  |
|  |  | Sl 06 | Sl | Sl | |  | Sl  (JQ286169) | Sl  (KC478779) | Sl  (JQ286162) |  |
|  | Ro | Sl 07 | Sl | Sl | |  | Sl  (JQ286169) | Sl  (KC478779) | Sl  (JQ799137) |  |
|  | Ff | Sl 08 | Sl | Sl | |  | Sl  (JQ286169) | Sl  (KC478779) | Sl  (JQ286158) |  |
|  |  | Sl 09 | Sl | Sl | |  | Sl  (JQ286169) | Sl  (KC478779) | Sl  (JQ286162) |  |
|  |  | Sl 10 | Sl | Sl | |  | Sl  (JQ286169) | Sl  (KC478779) | Sl  (JQ286158) |  |
|  |  | Sl 11 | Sl | Sl | |  | Sl  (JQ286169) | Sl  (KC478779) | Sl  (JQ286162) |  |
|  | Ra | Sl 12 | Sl | Sl | |  | Sl  (JQ286169) | Sl  (KC478779) | Sl  (JQ286159) |  |
|  |  | Sl 13 | Sl | Sl | |  | Sl  (JQ286169) | Sl  (KC478779) | Sl  (JQ286160) |  |
|  | Fp | Sl 14 | Sl | Sl | |  | **Rr**  (JQ286163) | **Rr**  (KC478782) | **Rr**  (JQ286150) |  |
|  |  | Sl 15 | Sl | Sl | |  | Sl  (JQ286169) | Sl  (KC478780) | Sl  (JQ286157) |  |
|  | Sv | Sl 16 | Sl | **Rr** | |  | Sl  (JQ286169) | Sl  (KC478779) | Sl  (JQ286157) |  |
|  |  | Sl 17 | Sl | Sl | |  | Sl  (JQ286169) | Sl  (KC478779) | Sl  (JQ286157) |  |
|  |  | Sl 18 | Sl | Sl | |  | Sl  (JQ286169) | Sl  (KC478779) | Sl  (JQ286157) |  |
| *S. squalus* | Sp | Ss 19 | Ss | Ss | |  | Ss  (JQ286166) | Ss  (KC478781) | Ss  (JQ286156) |  |
|  |  | Ss 20 | Ss | Ss | |  | Ss  (JQ286166) | Ss  (KC478781) | Ss  (JQ286156) |  |
|  | Fp | Ss 21 | Ss | Ss | |  | Ss  (JQ286166) | Ss  (KC478781) | Ss  (JQ286155) |  |
|  | Sv | Ss 22 | Ss | Ss | |  | **Sl**  (JQ286167) | **Sl**  (KC478779) | Ss  (JQ286154) |  |
| *T. muticellus* | Sp | Tm 23 | **Ss** | Tm | |  | Tm  (JQ286165) | Tm  (KC478783) | Tm  (JQ286153) |  |
|  |  | Tm 24 | Tm | Tm | |  | Tm  (JQ286165) | Tm  (KC478783) | Tm  (JQ799135) |  |
|  | Sv | Tm 25 | Tm | Tm | |  | Tm  (JQ286165) | Tm  (KC478783) | Tm  (JQ286152) |  |
| *R. rubilio* | Sp | Rr 26 | **Sl** | Rr | |  | Rr  (JQ286164) | Rr  (KC478782) | Rr  (JQ286151) |  |
|  | Ra | Rr 27 | Rr | Rr | |  | Rr  (JQ286164) | Rr  (KC478782) | Rr  (JQ286151) |  |
|  | Sv | Rr 28 | Rr | Rr | |  | Rr  (JQ286163) | Rr  (KC478782) | Rr  (JQ286150) |  |
