# Supplementary material for: Testing Species Delimitations in Four Italian Sympatric Leuciscine Fishes in the Tiber River: A Combined Morphological and Molecular Approach
Source: PLoS One. 2013 Apr 2;8(4):e60392. doi: 10.1371/journal.pone.0060392 (PMC3614999; doi:10.1371/journal.pone.0060392)
Supplement: Table S3 — Primers and protocols used for amplifications. (DOC) [file pone.0060392.s006.doc]

| **Gene** | **Primers**  **(Source)** | **PCR conditions** |
| --- | --- | --- |
| **Cyprinid formerly unknown nuclear Polymorphism**  **(Cyfun P)** | Cyp_un FLP1: 5’AAGTGGTGCATCGTGTTGTG3’ [39]  Cyp_unFL1R: 5’CAGCCTGAACAATCAAAACAG3’ [39] | 94ºC, 3 min - 35 cycles: 94ºC, 15 s; 55ºC, 20 s; 72º, 45 s - 72ºC, 7 min. |
| **Recombinant activating gene 1 (RAG1)** | RAG1F: 5’AGCTGTAGTCAGTAYCACAARATG3’ [50]  RAG9R: 5’GTGTAGAGCCAGTGRTGYTT3’ [50] | 95ºC, 3 min - 10 cycles: 95ºC, 45 s; 58ºC, 1.15 min.; 72º, 1.45 min., with reduction of 0.5ºC each annealing cycle (58ºC-53ºC) - 25 cycles: 95ºC, 45 s; 53ºC, 1.15 min.; 72º, 1.45 min. - 72ºC, 10 min. |
| **Cytochrome *b* (cytb)** | GluFor: 5’ACCADTGACNTGAAAAACCACCGTTG3’ [51]  H15915-Thr: 5’ACCTCCGATCTYCGGATTACAAGAC3’ [52] | 92ºC, 2 min. - 30 cycles: 92ºC, 15 s; 60ºC, 45 s; 72º, 1.50 min. - 72ºC, 8 min. |
