# Supplementary material for: Testing Species Delimitations in Four Italian Sympatric Leuciscine Fishes in the Tiber River: A Combined Morphological and Molecular Approach
Source: PLoS One. 2013 Apr 2;8(4):e60392. doi: 10.1371/journal.pone.0060392 (PMC3614999; doi:10.1371/journal.pone.0060392)
Supplement: Table S4 — Pairwise genetic distances used in PCOA analysis. (DOC) [file pone.0060392.s007.doc]

|  | *S. lucumonis* | | | | | | | | | | | | | | | | | | *S. squalus* | | | | *T. muticellus* | | | *R. rubilio* | | |
| --- | --- | --- | --- | --- | --- | --- | --- | --- | --- | --- | --- | --- | --- | --- | --- | --- | --- | --- | --- | --- | --- | --- | --- | --- | --- | --- | --- | --- |
|  | Sl01 | Sl02 | Sl03 | Sl04 | Sl05 | Sl06 | Sl07 | Sl08 | Sl09 | Sl10 | Sl11 | Sl12 | Sl13 | Sl14 | Sl15 | Sl16 | Sl17 | Sl18 | Ss19 | Ss20 | Ss21 | Ss22 | Tm23 | Tm24 | Tm25 | Rr26 | Rr27 | Rr28 |
| Sl01 | - |  |  |  |  |  |  |  |  |  |  |  |  |  |  |  |  |  |  |  |  |  |  |  |  |  |  |  |
| Sl02 | 0.001 | - |  |  |  |  |  |  |  |  |  |  |  |  |  |  |  |  |  |  |  |  |  |  |  |  |  |  |
| Sl03 | 0.002 | 0.001 | - |  |  |  |  |  |  |  |  |  |  |  |  |  |  |  |  |  |  |  |  |  |  |  |  |  |
| Sl04 | 0.001 | 0.000 | 0.001 | - |  |  |  |  |  |  |  |  |  |  |  |  |  |  |  |  |  |  |  |  |  |  |  |  |
| Sl05 | 0.002 | 0.001 | 0.000 | 0.001 | - |  |  |  |  |  |  |  |  |  |  |  |  |  |  |  |  |  |  |  |  |  |  |  |
| Sl06 | 0.002 | 0.001 | 0.000 | 0.001 | 0.000 | - |  |  |  |  |  |  |  |  |  |  |  |  |  |  |  |  |  |  |  |  |  |  |
| Sl07 | 0.000 | 0.000 | 0.001 | 0.000 | 0.002 | 0.001 | - |  |  |  |  |  |  |  |  |  |  |  |  |  |  |  |  |  |  |  |  |  |
| Sl08 | 0.001 | 0.002 | 0.001 | 0.002 | 0.001 | 0.001 | 0.001 | - |  |  |  |  |  |  |  |  |  |  |  |  |  |  |  |  |  |  |  |  |
| Sl09 | 0.002 | 0.001 | 0.000 | 0.001 | 0.000 | 0.000 | 0.001 |  | - |  |  |  |  |  |  |  |  |  |  |  |  |  |  |  |  |  |  |  |
| Sl10 | 0.001 | 0.002 | 0.001 | 0.002 | 0.001 | 0.001 | 0.001 | 0.000 | 0.001 | - |  |  |  |  |  |  |  |  |  |  |  |  |  |  |  |  |  |  |
| Sl11 | 0.002 | 0.001 | 0.000 | 0.001 | 0.000 | 0.000 | 0.001 | 0.001 | 0.000 | 0.001 | - |  |  |  |  |  |  |  |  |  |  |  |  |  |  |  |  |  |
| Sl12 | 0.004 | 0.003 | 0.002 | 0.003 | 0.002 | 0.002 | 0.003 | 0.003 | 0.002 | 0.003 | 0.002 | - |  |  |  |  |  |  |  |  |  |  |  |  |  |  |  |  |
| Sl13 | 0.002 | 0.001 | 0.000 | 0.001 | 0.001 | 0.000 | 0.002 | 0.001 | 0.000 | 0.001 | 0.000 | 0.002 | - |  |  |  |  |  |  |  |  |  |  |  |  |  |  |  |
| Sl14 | 0.081 | 0.081 | 0.081 | 0.081 | 0.082 | 0.081 | 0.082 | 0.081 | 0.081 | 0.081 | 0.081 | 0.082 | 0.082 | - |  |  |  |  |  |  |  |  |  |  |  |  |  |  |
| Sl15 | 0.001 | 0.000 | 0.001 | 0.000 | 0.002 | 0.001 | 0.001 | 0.002 | 0.001 | 0.002 | 0.001 | 0.003 | 0.002 | 0.082 | - |  |  |  |  |  |  |  |  |  |  |  |  |  |
| Sl16 | 0.001 | 0.000 | 0.001 | 0.000 | 0.001 | 0.001 | 0.000 | 0.002 | 0.001 | 0.002 | 0.001 | 0.003 | 0.001 | 0.081 | 0.000 | - |  |  |  |  |  |  |  |  |  |  |  |  |
| Sl17 | 0.001 | 0.000 | 0.001 | 0.000 | 0.001 | 0.001 | 0.000 | 0.002 | 0.001 | 0.002 | 0.001 | 0.003 | 0.001 | 0.081 | 0.000 | 0.000 | - |  |  |  |  |  |  |  |  |  |  |  |
| Sl18 | 0.001 | 0.000 | 0.001 | 0.000 | 0.001 | 0.001 | 0.000 | 0.002 | 0.001 | 0.002 | 0.001 | 0.003 | 0.001 | 0.081 | 0.000 | 0.000 | 0.000 | - |  |  |  |  |  |  |  |  |  |  |
| Ss19 | 0.054 | 0.054 | 0.053 | 0.054 | 0.053 | 0.053 | 0.054 | 0.053 | 0.053 | 0.053 | 0.053 | 0.054 | 0.053 | 0.077 | 0.054 | 0.054 | 0.054 | 0.054 | - |  |  |  |  |  |  |  |  |  |
| Ss20 | 0.054 | 0.054 | 0.053 | 0.054 | 0.053 | 0.053 | 0.054 | 0.053 | 0.053 | 0.053 | 0.053 | 0.054 | 0.053 | 0.077 | 0.054 | 0.054 | 0.054 | 0.054 | 0.000 | - |  |  |  |  |  |  |  |  |
| Ss21 | 0.054 | 0.054 | 0.053 | 0.054 | 0.054 | 0.053 | 0.055 | 0.053 | 0.053 | 0.053 | 0.053 | 0.055 | 0.054 | 0.077 | 0.055 | 0.054 | 0.054 | 0.054 | 0.001 | 0.001 | - |  |  |  |  |  |  |  |
| Ss22 | 0.044 | 0.044 | 0.043 | 0.044 | 0.044 | 0.043 | 0.045 | 0.043 | 0.043 | 0.043 | 0.043 | 0.045 | 0.044 | 0.075 | 0.045 | 0.044 | 0.044 | 0.044 | 0.008 | 0.008 | 0.009 | - |  |  |  |  |  |  |
| Tm23 | 0.087 | 0.088 | 0.087 | 0.088 | 0.087 | 0.087 | 0.087 | 0.086 | 0.087 | 0.086 | 0.087 | 0.089 | 0.087 | 0.092 | 0.089 | 0.088 | 0.088 | 0.088 | 0.091 | 0.091 | 0.091 | 0.089 | - |  |  |  |  |  |
| Tm24 | 0.088 | 0.089 | 0.088 | 0.089 | 0.088 | 0.088 | 0.088 | 0.087 | 0.088 | 0.087 | 0.088 | 0.090 | 0.088 | 0.093 | 0.090 | 0.089 | 0.089 | 0.089 | 0.092 | 0.092 | 0.093 | 0.090 | 0.001 | - |  |  |  |  |
| Tm25 | 0.086 | 0.087 | 0.086 | 0.087 | 0.087 | 0.086 | 0.087 | 0.008 | 0.086 | 0.085 | 0.086 | 0.089 | 0.087 | 0.092 | 0.088 | 0.087 | 0.087 | 0.087 | 0.089 | 0.089 | 0.090 | 0.087 | 0.002 | 0.003 | - |  |  |  |
| Rr26 | 0.080 | 0.080 | 0.080 | 0.080 | 0.080 | 0.080 | 0.080 | 0.080 | 0.080 | 0.080 | 0.080 | 0.081 | 0.080 | 0.002 | 0.080 | 0.080 | 0.080 | 0.080 | 0.077 | 0.077 | 0.077 | 0.075 | 0.091 | 0.092 | 0.091 | - |  |  |
| Rr27 | 0.080 | 0.080 | 0.080 | 0.080 | 0.080 | 0.080 | 0.080 | 0.080 | 0.080 | 0.080 | 0.080 | 0.081 | 0.080 | 0.002 | 0.080 | 0.080 | 0.080 | 0.080 | 0.077 | 0.077 | 0.077 | 0.075 | 0.091 | 0.092 | 0.091 | 0.000 | - |  |
| Rr28 | 0.081 | 0.081 | 0.081 | 0.081 | 0.082 | 0.081 | 0.082 | 0.080 | 0.081 | 0.081 | 0.081 | 0.082 | 0.082 | 0.000 | 0.082 | 0.081 | 0.081 | 0.081 | 0.077 | 0.077 | 0.077 | 0.075 | 0.092 | 0.093 | 0.092 | 0.002 | 0.002 | - |
