# Supplementary material for: Testing Species Delimitations in Four Italian Sympatric Leuciscine Fishes in the Tiber River: A Combined Morphological and Molecular Approach
Source: PLoS One. 2013 Apr 2;8(4):e60392. doi: 10.1371/journal.pone.0060392 (PMC3614999; doi:10.1371/journal.pone.0060392)
Supplement: Table S5 — Available sequences for RAG 1, cyt b (GenBank Accession Number) and combined based phylogenetic analyses. (DOC) [file pone.0060392.s008.doc]

| **Species** | **RAG1** | **cyt *b*** | **River** |
| --- | --- | --- | --- |
| *Squalius lucumonis* | HM560448 | HM560190 | Arno |
| *Squalius lucumonis* |  | AJ252817 | Tiber |
| *Squalius lucumonis* haplo Tib1 |  | AJ252818 | Tiber |
| *Squalius lucumonis* haplo Tib2 |  | AJ252819 | Tiber |
| *Squalius lucumonis* |  | AF421828 | Ombrone |
| *Squalius lucumonis* |  | HM560189 | Arno |
| *Squalius squalus* | HM560456 | HM560204 | Vipava |
| *Squalius squalus* | HM560457 | HM560205 | Arno |
| *Squalius squalus** |  | AJ252803 | Po |
| *Squalius squalus** |  | AJ252785 | Alento |
| *Squalius squalus** |  | AJ252798 | Drin |
| *Squalius squalus** |  | AJ252789 | Lake Skadar |
| *Squalius squalus*** |  | AF421792 | Po |
| *Squalius squalus* |  | JQ652368 | Bacchiglione |
| *Telestes muticellus* | HM560464 | HM560218 | Tiber |
| *Telestes muticellus* |  | AY509852 | Isonzo |
| *Telestes muticellus* |  | AY509853 | Ombrone |
| *Telestes muticellus* |  | AY509854 | Tiber |
| *Telestes muticellus* |  | AY509855 | Volturno |
| *Telestes muticellus* |  | AY509856 | Sabato |
| *Telestes muticellus**** |  | AY494739 | Sieve |
| *Telestes muticellus* |  | AY838934 | Ombrone |
| *Telestes muticellus* |  | HM560219 | Tiber |
| *Telestes muticellus* |  | JQ652296 | Bacchiglione |
| *Telestes muticellus* |  | JQ652305 | Bacchiglione |
| *Telestes muticellus* |  | JQ652306 | Bacchiglione |
| *Telestes muticellus* |  | JQ652308 | Bacchiglione |
| *Telestes muticellus* |  | JQ652309 | Bacchiglione |
| *Telestes muticellus* |  | JQ651583 | Bevera |
| *Telestes muticellus* |  | JQ651585 | Bevera |
| *Telestes muticellus* |  | JQ651588 | Bevera |
| *Telestes muticellus* |  | JQ651635 | Po |
| *Telestes muticellus* |  | JQ651639 | Po |
| *Telestes muticellus* |  | JQ651640 | Po |
| *Telestes muticellus* |  | JQ651641 | Po |
| *Telestes muticellus* |  | JQ651643 | Po |
| *Telestes muticellus* |  | JQ651647 | Po |
| *Rutilus rubilio* |  | FJ025061 | Tiber |
| *Rutilus rubilio* |  | FJ025067 | Farma |
| *Rutilus rubilio* |  | HM560166 | Arno |
| *Pseudorasbora parva* | HM560436 | HM560155 | Kizilirmak |

There reported as: * *S. cephalus* (Adriatic lineage), ** *S. cephalus cabeda*, ****Leuciscus muticellus*
